# The effects of different training programs on sleep and academic performance of senior high school boy students: a randomized controlled trial

**DOI:** 10.3389/fpsyg.2025.1579114

**Published:** 2025-04-16

**Authors:** Yueming Zhao, Guangxin Li, Zhikang Zou, Xin Zhang, Shilun Hou

**Affiliations:** ^1^College of Sports Medicine and Rehabilitation, Beijing Sport University, Beijing, China; ^2^Research Department of Air Force Military Medical University, Xi'an, China

**Keywords:** senior high school boy students, physical training, sleep, PSQI, academic performance

## Abstract

**Objective:**

To explore the effects of different physical training programs on the sleep of boy students in a senior high school, and to provide a reference for effectively improving the sleep level of students in a senior high school, as well as optimizing the physical training program.

**Methods:**

77 boys in 11th grade of a senior high school were randomly divided into four groups: Group I (traditional training), Group II (strength training), Group III (HIIT training), and Group IV (strength +HIIT training). At the points before and after the intervention, the Pittsburgh Sleep Quality Index (PSQI) and examination scores were measured as indicators of the sleep health and academic performance of students in senior high school, and the intervention effects of different schemes were compared.

**Results:**

(1) After the intervention, the PSQI global score of Group II, Group III, and Group IV was significantly lower than that of Group I (*p* < 0.001, *p* = 0.004, *p* = 0.01, respectively), and the PSQI global score (*p* < 0.001, *p* = 0.02, *p* < 0.001, respectively), sleep latency (*p* = 0.008, *p* = 0.003, *p* = 0.005, respectively) and sleep duration (*p* < 0.001, *p* = 0.005, *p* = 0.003, respectively) were significantly lower than those before intervention; (2) After intervention, the score of sleep disturbances of Group IV was significantly lower than that before intervention (*p* = 0.02); (3) After the intervention, academic performance among the four groups show no significance (*p* = 0.886 > 0.05), while Group IV was significantly higher than that before intervention (*p* = 0.047).

**Conclusion:**

Compared with traditional training programs, strength training, HIIT and strength+HIIT training programs have a stabilizing and improving effect on the sleep health level of senior high school boy students, mainly reflected by reducing sleep latency and increasing sleep duration. All four training programs can improve academic performance, while the strength+HIIT training program produced a more significant effect.

## 1 Introduction

Sleep is an important physiological process (Baranwal et al., 2023; Sletten et al., 2023; Lim et al., 2023). About one-third of a person’s life is spent in sleep (Vorster et al., 2024). During sleep, the body completes the elimination of fatigue, the response and integration of the immune system, and the consolidation of memory (Miletínová and Bušková, 2021). It can be seen that sleep is important in the process of life. The senior high school stage is an important turning point in life. A large number of studies have shown that high school students are in adolescence, during which they have become one of the important groups of sleep deprivation due to various physiological and psychological complex factors (Liu et al., 2024; Kansagra, 2020; Xu et al., 2012; Mak et al., 2012). They are faced with many problems, such as the pressure of academics and examination, the change of identity, the construction of world outlook and values, the coexistence and running-in with others and the environment, which may produce relatively higher pressure (Gedda-Muñoz et al., 2023), resulting in poor sleep and even decreased academic performance (Liu et al., 2024). Therefore, in the cultivation process of high school students, we should pay attention to students’ sleep, and ameliorate and improve students’ sleep health.

Previous studies have shown a close relationship between poor sleep quality and physical inactivity (Visier-Alfonso et al., 2022; Wang et al., 2016; Spruit et al., 2016; Yeatts et al., 2017; Philippot et al., 2022) while there is a certain correlation between physical activity and students’ academic performance (Mak et al., 2012; Visier-Alfonso et al., 2022; Wang et al., 2016; Spruit et al., 2016). It is inferred that sleep quality will affect the academic level of adolescents (Spruit et al., 2016). Studies have shown that cardiorespiratory fitness and strength training are regulators of stress (Yeatts et al., 2017, Philippot et al., 2022). Strength training and aerobic training can improve sleep by improving mood state (Philippot et al., 2022; Carek et al., 2011; Vorster et al., 2024), interpersonal relationships (Smith et al., 2018), increasing energy consumption (Mendelson et al., 2016), and regulating the endocrine system (Uchida et al., 2012; Richards et al., 2011). In addition, aerobic training and strength training help to enhance physical fitness, regulate mood, relieve fatigue, and prevent mental illness, thereby indirectly improving sleep quality (Taylor et al., 2021; Vartanian et al., 2018; Guo et al., 2021; Albrektsen et al., 2021). Therefore, physical training has an important impact on sleep quality.

Despite the attention that has been given to exercise interventions for adolescents to improve sleep (Jurado-Fasoli et al., 2020; Longlalerng et al., 2021) and academic performance (Zhang et al., 2023; Robinson et al., 2023), few studies have focused specifically on males during adolescence - a critical period marked by rapid physiological maturation (e.g., testosterone-driven muscle growth) and high academic stress. Existing literature disproportionately emphasizes female participants or mixed-gender cohorts, despite evidence that boys exhibit different responses to physical activity (Landen et al., 2023). This gap limits the development of targeted interventions for male students, who also globally experience lower sleep quality (Krishnan and Collop, 2006; Safarzade and Tohidinik, 2019) and higher academic disengagement compared to their female peers (Hysing et al., 2016; Yunus et al., 2021). In a survey of 7,798 adolescent students (16–19 years old) included in the study, Hysing et al. found that male students had significantly lower academic achievement (grade point average) than female students with equally poor sleep quality (Hysing et al., 2016). However, as a special group of high school students, there are few studies on their sleep-related issues. Whether physical training can ameliorate or improve the sleep problems of high school boy students is worthy of further discussion.

To test which training program would be more effective in improving or increasing sleep and academic performance in male adolescent high school students. We hypothesized that strength training, HIIT training, and combined exercise would all play better results compared to traditional training, with combined (HIIT+ST) exercise achieving better results. Therefore, this study applied systematic physical training to high school students for the first time and explored the effects of four different physical training programs on high school students’ sleep and academic performance. It provides a reference for effectively regulating and ameliorating their sleep status and improving academic performance. At the same time, it will provide a theoretical basis for optimizing physical training programs.

## 2 Methods

### 2.1 Participants

A convenience sample of 77 students in 11th grade from a public senior high school in Sichuan province, western China, was selected for this study, and their characteristics are shown in Table 1. Participants were all apparently healthy, as defined by their enrollment in PE class, and able to participate in regular exercise. No other inclusion or exclusion criteria were applied. For almost all boarding students, the daily school schedule is similar. The day typically began with an optional morning exercise and reading between 6:50 AM and 7:20 AM. Formal morning classes started at 8:10 AM and ended at 12:00 AM, with physical activity during recess from 9:50 to 10:20, followed by a 2.5-h lunch and noon break. During the noon break, all students remained at school and had the opportunity to study or take brief naps after lunch. Afternoon classes resumed at 2:35 PM and ended at 6:10 PM. The students typically had dinner from 6:10 PM to 6:45 PM. 3 optional evening studies in school ended by 10:00 PM.

**Table 1:** Participants’ physical characteristics.

Characteristics	I (*n* = 20)	II (*n* = 19)	III (*n* = 19)	IV (*n* = 19)
Age, year	16.75 ± 0.55	16.32 ± 0.48	16.84 ± 0.38	16.79 ± 0.54
Height, cm	172.49 ± 4.79	171.07 ± 4.22	171.92 ± 4.53	171.66 ± 5.03
Weight, kg	64.02 ± 7.19	60.53 ± 4.18	65.83 ± 4.83	62.51 ± 6.68
BMI, kgm^−2^	21.72 ± 2.44	20.94 ± 2.30	22.27 ± 1.25	21.72 ± 2.36
Body fat, kg	15.00 ± 2.56	15.42 ± 6.05	14.62 ± 3.30	16.23 ± 2.18
Lean body mass, kg	35.38 ± 3.17	33.42 ± 5.07	36.41 ± 3.44	36.86 ± 2.77
Truck muscle, kg	24.83 ± 2.22	24.27 ± 1.53	25.55 ± 2.41	25.86 ± 1.95

Participants were informed of the study purposes, procedures, and potential risks, and provided written information consent before commencing the study, and the reporting follows the general guidelines described in the Consolidated Standards of Reporting Trials (CONSORT) 2010 statement. The study was formally approved by the Beijing Sports University Human Ethics Committee (Approval No.:2024352H) and conducted according to the Declaration of Helsinki. Before starting the present study, a power analysis was performed (G*Power 3.1, Heinrich-Heine-Universität, Düsseldorf, Germany) to calculate the adequate sample size (*F*-test, effect size = 0.25, *α* error = 0.05, power = 0.95) (Faul et al., 2009). According to this calculation, 76 participants were required. When considering a 10% dropout and the actual situation, the sample size was included in a total of 90.

### 2.2 Instruments

#### 2.2.1 Sleep patterns

Participants completed the Chinese version of the Pittsburgh Sleep Quality Index (PSQI) which contains 19 self-evaluation items and 5 other-evaluation items (Buysse et al., 1989; Liu and Tang, 1996). The PSQI was initially developed by Buysse et al. (1989) and is a self-report assessment tool that evaluates sleep quality over 1 month. A global score and seven component scores can be derived from the scale. The component scores are the following: Subjective sleep quality, sleep latency, sleep duration, sleep efficiency, sleep disturbances, use of sleeping medications, and daytime dysfunction. The seven component scores are summed to obtain a global score ranging from 0 to 21. Based on the original study, scores larger than 5 indicate poor sleep quality, which yielded a specificity of 86.5% and a sensitivity of 89.6% in distinguishing good and poor sleepers (Buysse et al., 1989) across several populations (Carpenter and Andrykowski, 1998), including the elderly (Buysse et al., 1989). Higher scores on each component indicate poorer sleep. This scale has been translated into many different languages and is a well-established scale with acceptable psychometric properties among numerous clinical and non-clinical populations (Nakajima et al., 2014; Ait-Aoudia et al., 2013; Manzar et al., 2015).

#### 2.2.2 Academic performance

Academic performance was measured with exam scores. The test scores before training are used as the pre-test academic performance, and the test scores after training are used as the post-test academic performance.

### 2.3 Procedure

#### 2.3.1 Randomization and allocation

A longitudinal, randomized controlled trial was conducted. The adolescents were assigned to 4 groups: Group I (traditional training, RG + ET), Group II (strength training, RG + ST), Group III (HIIT training, HIIT+ET) or Group IV (HIIT+ST), through random digital method. The study will include a random assignment of volunteers into three groups. The volunteers will be not blinded and will be aware of which training mode will be used. The examiners will not know about the study. The statistician will also be blinded to intervention allocation. The data will be coded in an unrecognizable manner, with no information indicating which group a single participant will be assigned to. Eventually, 77 adolescents were allocated between a Group I (*n* = 20), Group II (*n* = 19), Group III (*n* = 19) and Group IV (*n* = 19).

Four groups of students participated in a 12-week physical training program during the same academic year and in the same conditioned environment as each group’s physical training program. The sleep quality scores and academic performance of all students were measured before and after the intervention to compare the effects of different training programs on the sleep and academic performance of high school students.

**Figure 1:**
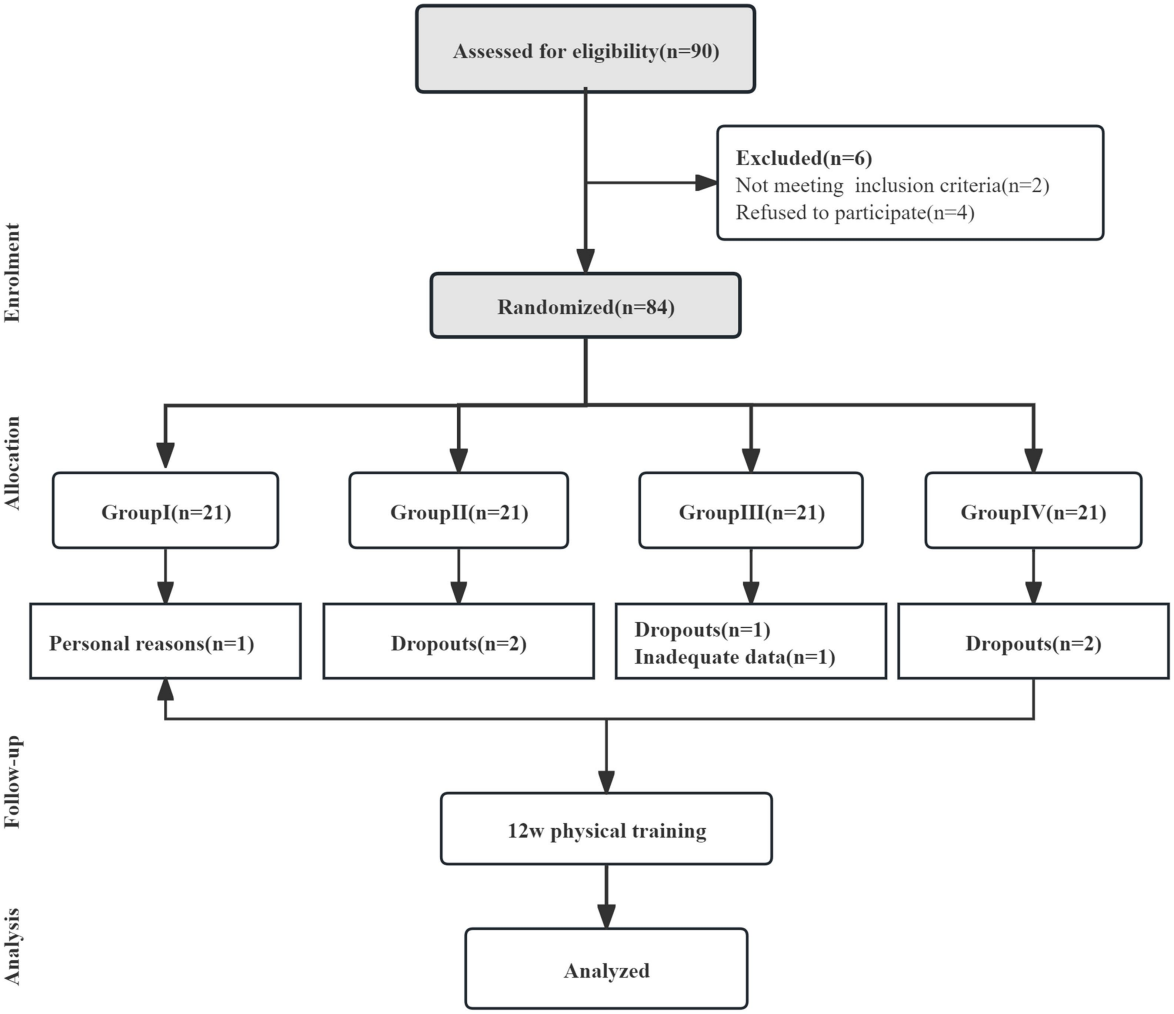
The flow-chart of this study.

School officials and adolescents at this school permitted us to administer the survey and access the exam scores. Two classes were randomly selected from the school (n = 90), and all student participation was entirely voluntary. The questionnaire was distributed for the first time before students began training in October 2024. A total of 84 questionnaires were distributed to students during class and 84 completed questionnaires were returned, yielding a response rate of 100%. During the training period, 7 people withdrew or did not meet the requirements due to various reasons. In January 2025, the second distribution was carried out after the training. A total of 77 questionnaires were distributed to students during class and 77 completed questionnaires were returned, yielding a response rate of 100%. Questionnaires were distributed, completed, and returned within the time of one class. In addition, the examination scores of the students who completed the questionnaires were obtained from the school, including entrance examination scores and final examination scores. Importantly, no injury or adverse events occurred during the 12-week training. The flow-chart of the study is presented in Figure 1.

#### 2.3.2 Physical training protocol during recess

There are two main forms of exercise during recess: (1) Radio gymnastics (RG), mainly carries out the Ninth Set of radio gymnastics exercises that follow music for 20 min, 6 times a week. (2) HIIT, mainly to follow the Tabata music *in situ* running, jumping, squatting, and other high-intensity interval training, each training 20 min, 6 times a week. Specifically as follows: HIIT training sessions were designed for a session length of approximately 20 min. Each training session started with 5 min warm-up using brisk walking, followed by three rounds of 10 exercises, each performed for 20 s and followed by 10 s of rest, following the timing ratio of Tabata et al. (1996). The specific scheme is: the adolescents in the group III and IV were assigned to HIIT 6 times per week, for a total of 12 weeks. Previous research showed that short rest between exercise bouts is associated with maximized metabolic impact (Haltom et al., 1999). The protocol focused on training using body weight (no equipment needed) and aimed to incorporate a combination of aerobic exercises, strengthening exercises for the upper and lower body, and core stability exercises for beginners. A detailed description of the HIIT program including the exercise description, type of exercise, and target muscle group is presented in Supplementary material 1. For example, one session selected ten exercises are: (1) jumping jack, (2) wall sit, (3) push-up, (4) abdominal crunch, (5) squat, (6) plank, (7) lunge, (8) side plank (right side), (9) side plank (left side), and (10) high knees running and so on. The selected exercises included in our program showed accessibility, practicality, and time-saving for individuals who previously felt they did not have enough resources or time to exercise (Klika and Jordan, 2013).

#### 2.3.3 Physical education

Physical education training mainly for two forms of exercise: (1) Endurance training (ET), mainly for 3,000 m endurance running and free activities, each session 90 min, 2 times a week; (2) strength training (ST), the first 4 weeks to establish the correct basic action mode, the development of flexibility, upper and lower limb muscle endurance, core stability as the main purpose; the main purpose of 5-10th weeks was to develop the muscle circumference and core strength of the upper and lower limbs. The main purpose of 11-12th weeks is to develop maximum muscle strength and continue to strengthen core strength. Each training is 90 min and 2 times a week. The specific scheme is: the adolescents in the group II and IV were assigned to strength training 2 times per week, for a total of 12 weeks. Each session lasted for approximately 90 min, including 15 min of warm-up, 60 min of formal training, and 15 min of stretching. The training session consisted of about 8–10 training exercises, each of which was repeated in 3 sets of 10–12 repetitions, with a 1-min rest interval between sets and exercises. The exercises were performed in an alternated-by-segment fashion as follows; squat, deadlift, leg extension, hamstring curls, bench press, biceps curl, rowing, lat pull-down, and so on (Lloyd et al., 2014). A detailed description of the strength training database including the type of exercise, and target muscle group is presented in Supplementary material 2.

Intensity was based on a predicted one-repetition maximum (1RM), as outlined in the American College of Sports Medicine guidelines (Franklin et al., 2010). Assessment of 1RM was completed at baseline and then re-assessed every 4 weeks, to account for any strength gains. Resistive load increased fortnightly from 75 percent 1RM over the initial 12 weeks, then to 85 percent 1RM for the last 4 weeks. Participants were to be removed from the study if they missed three consecutive training sessions or 10 percent of the training sessions over the 12 weeks.

### 2.4 Data analysis

All statistical analyses were conducted using IBM SPSS Statistics version 26.0 (IBM Corp., Armonk, NY). The Shapiro–Wilk Test was used to test the normality of the data. The series that conform to the normal distribution are expressed in the form of means and standard deviations (SD), and the series that do not conform to the normal distribution are expressed in the form of median and quartile (Q). A two-way repeated ANOVA, a one-way analysis of variance, and a paired T-test were used to determine significant differences between the groups. If the conditions were not met, the Wilcoxon signed-rank test was used for intra-group comparison, and the Kruskal-Wallis H test was used to compare the differences between groups. The significance level was defined as *p* < 0.05.

## 3 Results

### 3.1 PSQI global score

**Figure 2:**
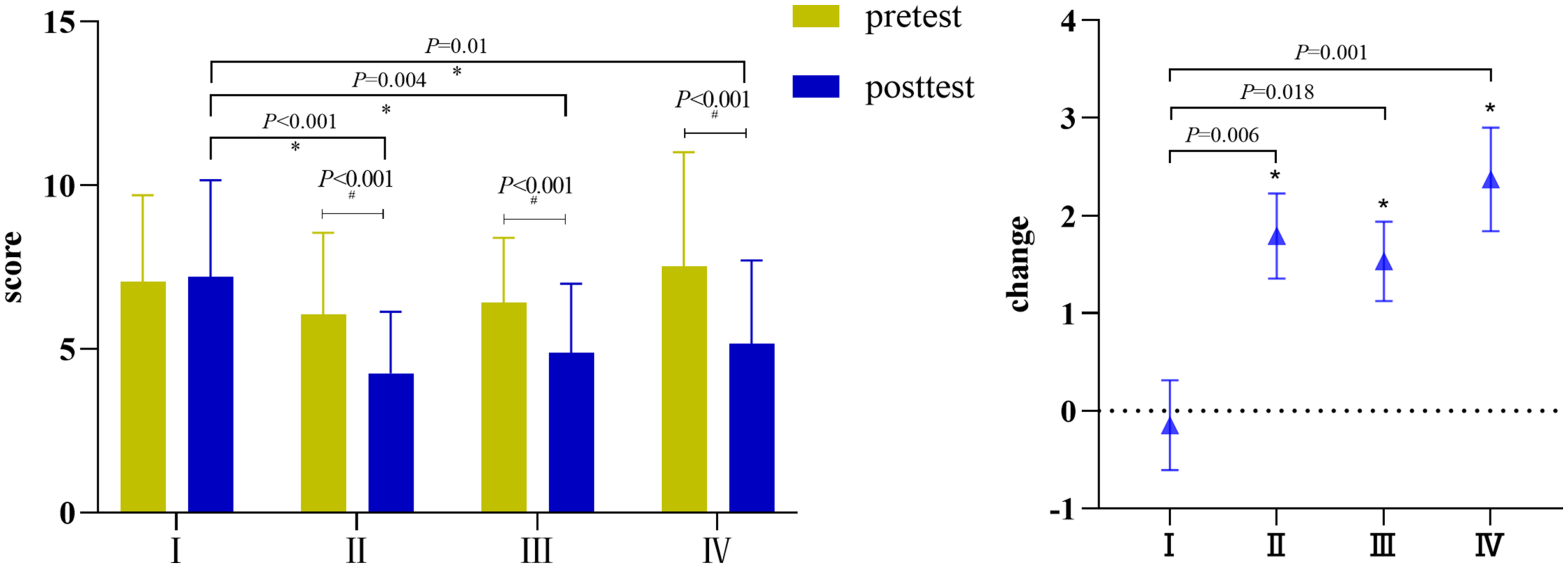
PSQI global score and change in each group.

The PSQI global scores of pre and post-intervention for all groups are shown in Table 2 and Figure 2. There was a significant interaction between the four groups of students’ PSQI global score time × group (η^2^ = 0.187, *p* = 0.002 < 0.05, *F* = 5.614), and the main effect of training group (η^2^ = 0.187, *p* = 0.061, *F* = 2.561) was not significant. The main effect of training time (η^2^ = 0.33, *p* < 0.001, *F* = 35.984) was significant. A pairwise comparison showed no significant difference between the four groups before training. Compared with before training, there was no substantial change in the PSQI global score of Group I after training (95% CI–1.052–0.751, *p* = 0.741 > 0.05). In contrast, the PSQI global scores of the other three groups were significantly lower, Group II (95%CI 0.864–2.715, *p* < 0.001), Group III (95%CI 0.671–2.382, *p* = 0.02), Group IV (95%CI 1.447–3.294, *p* < 0.001). Additionally, the PSQI global scores in Group II (95%CI 1.396–4.487, *p* < 0.001), Group III (95%CI 0.764–3.864, *p* = 0.004), and Group IV (95%CI 0.501–3.583, *p* = 0.01) were significantly lower than those in Group I.

**Table 2:** PSQI scores before and after intervention in each group.

Group	Pretest		Protest		∆	
	Mean	SD	Mean	SD	Mean (Median)	SD(Q)
I (*n* = 20)	7.05	2.65	7.20	2.95	−0.15 (0)	2.06 (2.00)
II (*n* = 19)	6.05	2.51	4.26^#*^	1.88	1.79 (1.00)^*^	1.90 (4.00)
III (*n* = 19)	6.42	1.98	4.89^#*^	2.11	1.53 (1.00)^*^	1.78 (3.00)
IV (*n* = 19)	7.53	3.49	5.16^#*^	2.54	2.37 (2.00)^*^	2.31 (2.00)

Interaction between the group and time: η^2^ = 0.18, *p* = 0.002, *F* = 5.614. #Significance in pre and post, *p* < 0.05. *Significance between Group I and other groups, *p* < 0.05. ∆: Pretest-posttest.

After training, the Kruskal-Wallis H test showed that there was a significant difference in the reduction of PSQI global scores between the groups (*H* = 13.326, *p* = 0.004). The Bonferroni method was used to correct the post-hoc pairwise comparison of the significance level. It was found that the reduction values of Group II, Group III and Group IV were significantly higher than those of Group I (*p* = 0.006, *p* = 0.018, *p* = 0.001, respectively), while there was no significant difference between Group II, Group III and Group IV. The order of change value was Group IV > Group II > Group III > Group I.

### 3.2 Sleep quality

The Wilcoxon signed rank test revealed, compared with before training, non-significance change in sleep quality scores of students in each group, Group I (*Z* = –1.730, *p* = 0.084), Group II (*Z* = –1.342, *p* = 0.180), Group III (*Z* = –0.378, *p* = 0.705), group IV (*Z* = –1.667, *p* = 0.096).

After training, the Kruskal-Wallis H test showed that there was no significant difference in the reduction of sleep quality scores between the groups (H = 2.934, *p* = 0.402). As shown in Table 3.

**Table 3:** Sleep quality scores before and after intervention in each group.

Group	Pretest		Protest		∆	
	Mean (Median)	SD(Q)	Mean (Median)	SD(Q)	Mean (Median)	SD(Q)
I (*n* = 20)	1.50 (1.00)	0.61 (1.00)	1.2 (1.00)	0.62 (1.00)	0.30 (0)	0.73 (1.00)
II (*n* = 19)	1.11 (1.00)	0.57 (0)	0.95 (1.00)	0.62 (0)	0.16 (0)	0.50 (0)
III (*n* = 19)	1.16 (1.00)	0.69 (1.00)	1.21 (1.00)	0.63 (1.00)	−0.05 (0)	0.62 (0)
IV (*n* = 19)	1.42 (1.00)	0.77 (1.00)	1.16 (1.00)	0.83 (1.00)	0.26 (0)	0.65 (1.00)

∆: Pretest-posttest.

### 3.3 Sleep latency

The Wilcoxon signed rank test revealed, compared with before training, non-significance change in the score of sleep latency after training in group I (*Z* = –0.302, *p* = 0.763), while the scores of sleep latency after training in the other three groups were significantly reduced, group II (*Z* = –2.673, *p =* 0.008), Group III (*Z* = –2.972, *p* = 0.003), Group IV (*Z* = –2.84, *p* = 0.005), that showed sleep latency was improved.

After training, the Kruskal-Wallis H test showed that there was statistical significance between the scores of sleep latency in each group (H = 9.307, *p* = 0.025). The Bonferroni method was used to correct the post-hoc pairwise comparison of the significance level. It was found that the reduction values of Group II, Group III, and Group IV were significantly higher than those of Group I (*p* = 0.023, *p* = 0.01, *p* = 0.011, respectively), while there was no significant difference between Group II, Group III, and Group IV. As shown in Table 4.

**Table 4:** Sleep latency scores before and after intervention in each group.

Group	Pretest		Protest		∆	
	Mean (Median)	SD (Q)	Mean (Median)	SD (Q)	Mean (Median)	SD (Q)
I (*n* = 20)	1.45 (1.00)	0.83 (1.00)	1.50 (1.00)	0.76 (1.00)	−0.05 (0)	0.76 (2.00)
II (*n* = 19)	1.47 (1.00)	0.70 (1.00)	0.95 (1.00)^#^	0.71 (0)	0.53 (1.00)^*^	0.70 (1.00)
III (*n* = 19)	1.63 (2.00)	0.83 (2.00)	1.00 (1.00)^#^	0.67 (1.00)	0.63 (1.00)^*^	0.68 (1.00)
IV (*n* = 19)	1.84 (2.00)	1.12 (2.00)	1.26 (1.00)^#^	0.87 (1.00)	0.58 (1.00)^*^	0.69 (1.00)

#Significance in pre and post, *p*<0.05. *Significance between Group I and other groups, *p*<0.05. ∆: Pretest-posttest.

### 3.4 Sleep duration

The Wilcoxon signed rank test revealed, compared with before training, non-significance change in the sleep duration of group I after training (*Z* = –1.134, *p* = 0.257), while the sleep duration scores of the other three groups after training were significantly reduced, Group II (*Z* = –3.606, *p* < 0.001), Group III (*Z* = –2.840, *p* = 0.005), Group IV (*Z* = –2.972, *p* = 0.003), that showed sleep duration was improved.

After training, the Kruskal-Wallis H test showed that the reduction value of sleep duration scores in each group was statistically significant (H = 8.578, *p* = 0.035). The Bonferroni method was used to correct the post-hoc pairwise comparison of the significance level. It was found that the reduction values of Group II, Group III, and Group IV were significantly higher than those of Group I (*p* = 0.007, *p* = 0.034, *p* = 0.032, respectively), while there was no significant difference between Group II, group III, and Group IV. As shown in Table 5.

**Table 5:** Sleep duration scores before and after intervention in each group.

Group	Pretest		Protest		**∆**	
	Mean (Median)	SD (Q)	Mean (Median)	SD (Q)	Mean (Median)	SD (Q)
I (*n* = 20)	1.10 (1.00)	0.55 (0)	0.95 (1.00)	0.51 (0)	0.15 (0)	0.59 (1.00)
II (*n* = 19)	0.84 (1.00)	0.38 (0)	0.16 (0)^#^	0.38 (0)	0.68 (1.00)^*^	0.48 (1.00)
III (*n* = 19)	1.05 (1.00)	0.52 (0)	0.47 (0)^#^	0.61 (1.00)	0.58 (1.00)^*^	0.69 (1.00)
IV (*n* = 19)	1.00 (1.00)	0.67 (0)	0.37 (0)^#^	0.50 (1.00)	0.63 (1.00)^*^	0.68 (1.00)

#Significance in pre and post, *p*<0.05. *Significance between Group I and other groups, *p*<0.05. ∆: Pretest-posttest.

### 3.5 Sleep efficiency

The Wilcoxon signed rank test revealed, compared with before training, the sleep efficiency scores of each group after training were not statistically significant, and the sleep efficiency was not significantly improved. Group I (*Z* = –0.333, *p* = 0.739), Group II (*Z* = –1.414, *p* = 0.157), Group III (*Z* = –1.732, *p* = 0.083), Group IV (*Z* = –1.890, *p* = 0.059).

After training, the Kruskal-Wallis H test showed that there was no significant difference in the reduction value of sleep efficiency scores among the groups (H = 2.745, *p* = 0.433). As shown in Table 6.

**Table 6:** Sleep efficiency scores before and after intervention in each group.

Group	Pretest		Protest		∆	
	Mean (Median)	SD (Q)	Mean (Median)	SD (Q)	Mean (Median)	SD (Q)
I (*n* = 20)	0.25 (0)	0.55 (0)	0.30 (0)	0.57 (1.00)	−0.05 (0)	0.69 (0)
II (*n* = 19)	0.11 (0)	0.32 (0)	0 (0)	0 (0)	0.11 (0)	0.32 (0)
III (*n* = 19)	0.16 (0)	0.38 (0)	0 (0)	0 (0)	0.16 (0)	0.38 (0)
IV (*n* = 19)	0.37 (0)	0.36 (0)	0 (0)	0 (0)	0.37 (0)	0.76 (0)

∆: Pretest-posttest.

### 3.6 Sleep disturbances

The Wilcoxon signed rank test revealed, compared with before training, the scores of sleep disturbances in each group after training were not statistically significant, and the sleep disturbances were not significantly improved. Group I (*Z* = –0.00, *p* = 1), Group II (*Z* = –0.333, *p* = 0.739), Group III (*Z* = –1.732, *p* = 0.083), Group IV (*Z* = –2.333, *p* = 0.02).

After training, the Kruskal-Wallis H test showed that there was no significant difference in the reduction value of sleep disturbance scores among the groups (H = 4.133, *p* = 0.247). As shown in Table 7.

**Table 7:** Sleep disturbances scores before and after intervention in each group.

Group	Pretest		Protest		**∆**	
	Mean (Median)	SD (Q)	Mean (Median)	SD (Q)	Mean (Median)	SD (Q)
I (*n* = 20)	1.10 (1.00)	0.31 (0)	1.10 (1.00)	0.64 (1.00)	0 (0)	0.65 (0)
II (*n* = 19)	1.05 (1.00)	0.62 (0)	1.00 (1.00)	0.67 (0)	0.05 (0)	0.71 (1.00)
III (*n* = 19)	1.16 (1.00)	0.38 (0)	1.00 (1.00)	0 (0)	0.16 (0)	0.38 (0)
IV (*n* = 19)	1.16 (1.00)	0.50 (0)	0.79 (1.00)^#^	0.42 (0)	0.37 (0)	0.60 (1.00)

#Significance in pre and post, *p*<0.05. *p*<0.05. ∆: Pretest-posttest.

### 3.7 Daytime dysfunction

The Wilcoxon signed rank test revealed, compared with before training, the scores of daytime dysfunction in each group after training were not statistically significant, and sleep disorders were not significantly improved, Group I (*Z* = –0.378, *p* = 0.705), Group II (*Z* = –0.816, *p* = 0.414), Group III (*Z* = –0.973, *p* = 0.331), Group IV (*Z* = –1.134, *p* = 0.257).

After training, the Kruskal-Wallis H test showed that there was no significant difference in the reduction values of daytime dysfunction scores among the students in each group (H = 1.591, *p* = 0.661). As shown in Table 8.

**Table 8:** Daytime dysfunction scores before and after intervention in each group.

Group	Pretest		Protest		∆	
	Mean (Median)	SD (Q)	Mean (Median)	SD (Q)	Mean (Median)	SD (Q)
I (*n* = 20)	1.65 (1.5)	0.99 (2.00)	1.70 (2.00)	0.98 (2.00)	−0.05 (0)	0.83 (0)
II (*n* = 19)	1.32 (1.00)	0.89 (1.00)	1.21 (1.00)	0.63 (1.00)	0.11 (0)	0.57 (0)
III (*n* = 19)	1.26 (1.00)	0.87 (1.00)	1.05 (1.00)	0.85 (2.00)	0.21 (0)	0.92 (1.00)
IV (*n* = 19)	1.63 (2.00)	0.83 (1.00)	1.42 (1.00)	0.90 (1.00)	0.21 (0)	0.79 (0)

*p<0.05. ∆: Pretest-posttest.*

### 3.8 Academic performance

There was no significant interaction between the four groups of students’ academic performance time×group (η^2^ = 0.01, *p* = 0.886 > 0.05, *F* = 0.242), and the main effect of training group (η^2^ = 0.992, *p* = 0.896 > 0.05, *F* = 0.2) was not significant. The main effect of training time (η^2^ = 0.079, *p* = 0.015 < 0.05, *F* = 6.271) was significant.

**Figure 3:**
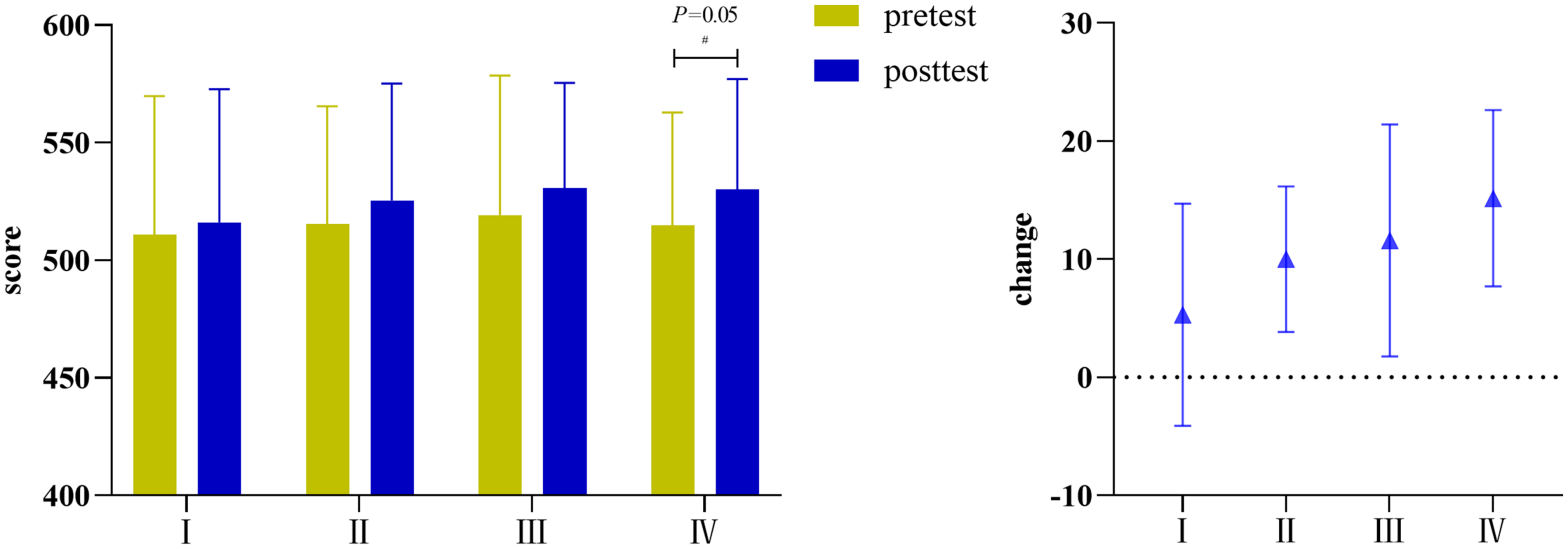
Academic performance scores and change in each group.

Compared with before training, there was no significant change in the academic performance scores of Group I (MD = 5.28, 95%CI 14.45–25.00, *p* = 0.582), Group II (MD = 10.00, 95%CI–2.95–22.95, *p* = 0.122) and Group III (MD = 11.58, 95%CI–9.06–32.22, *p* = 0.254) after training, while Group IV showed significant improvement (MD = 15.16, 95%CI –0.53–30.85, *p =* 0.05). The order of change value was Group IV > Group III > Group II > Group I. As shown in Table 9 and Figure 3.

**Table 9:** Academic performance scores before and after intervention in each group.

Group	Pretest		Posttest		∆	
	Mean	SD	Mean	SD	Mean	SD
I (*n* = 20)	510.90	58.84	516.17	56.65	5.30	42.11
II (*n* = 19)	515.42	50.14	525.42	49.75	10.00	26.88
III (*n* = 19)	519.21	59.40	530.79	44.61	11.58	42.82
IV (*n* = 19)	514.95	47.93	530.11^#^	46.96	15.16	32.55

Interaction between the group and time: η^2^ = 0.01, *p* = 0.886, *F* = 0.242. #Significance in pre and post, *p*<0.05. *p*<0.05. ∆: Pretest-posttest.

## 4 Discussion

This study explored the effects of different physical training programs on the overall sleep quality (including sleep quality, total sleep time, sleep disorder, sleep latency, sleep efficiency, use of sleeping medication, and daytime dysfunction) and academic performance of high school students. The sleep and academic benefits of the intervention are mainly reflected in the improvement of the overall sleep quality level, sleep time, sleep efficiency and other components, and the improvement of academic performance. Namely, physical training can not only effectively enhance and regulate the poor sleep status of senior students, improve sleep level, reduce life pressure, but also help to improve physical fitness and academic performance.

### 4.1 Effects of different physical training programs on the overall level of sleep quality

Due to the high school students are in adolescence, the period of psychological fluctuation is larger, and in the face of academic pressure, examination pressure, bad biological rhythm, and other problems, easy to appear obvious emotional changes, which affect sleep, daily life, and even academic performance (Astill et al., 2013; Qu et al., 2024). At the same time, studies have found that with the increase in grades, the overall level of sleep and the level of each component of high school students show a downward trend (Huang et al., 2010; Zhou et al., 2012; Chen et al., 2015). Therefore, regulating and improving the level of sleep quality is very important for high school students. The study found that the overall sleep level of high school students is closely related to their level of physical activity. The incidence of sleep disorders in high school students who exercise regularly is significantly lower than that of students who do not exercise regularly (Mahfouz et al., 2020; Richardson et al., 2017). Exercise can regulate body temperature (Shin et al., 2013), neuro-endocrine (Morris et al., 2012), circadian rhythm (Richardson et al., 2017), mood state (Konjarski et al., 2018), etc., so as to promote the occurrence of sleep and the maintenance of the sleep process, and improve sleep disorders.

In this study, after 12 weeks of physical training intervention, the PSQI global score of students who underwent strength training, HIIT training, and strength+HIIT training was significantly lower than that of students who underwent traditional training. It has a positive effect on improving the sleep quality of high school students and reducing the occurrence of sleep disorders, which is consistent with the existing research results (Mendelson et al., 2016; Richardson et al., 2017). Although the traditional training program has a clear way of exercise, it does not carry out specific load structure design, and the high school students may have physical and psychological adaptive changes (Hughes et al., 2018). As a result, the traditional training program has no obvious physical and psychological effects on the high school students, resulting in no significant improvement in sleep levels.

Through the PSQI score, the individual’s subjective feeling of sleep can reflect the degree of sleep disorder and sleep quality of high school students. Exercise can trigger various mechanisms to regulate and improve the subjective overall sleep quality. A large number of studies have found that strength training, HIIT and strength+HIIT training can effectively improve the overall subjective sleep quality of obese or chronic sleep disorders in middle-aged people, sedentary people, and cancer patients. Similarly, Mendelson et al. (2016) found that 12 weeks of aerobic training combined with strength training exercise intervention significantly improved the overall sleep quality (sleep efficiency, sleep time) of adolescents, which was consistent with this study. In addition, Amos et al. (2019) found that high-intensity exercise intervention for 4 weeks in adolescents with sleep problems can increase their sleep time, improve sleep efficiency, and reduce daytime sleepiness.

The results showed that the effects of strength training, HIIT, and strength+HIIT training on the overall sleep quality of high school students were consistent, but there was no significant difference between the overall effects of the training program. The study found that high-intensity aerobic training combined with strength training and high-intensity aerobic training alone were better than low-intensity aerobic training in improving the sleep quality of patients with adjuvant chemotherapy, and there was no difference between the two (Courneya et al., 2014). Both HIIT and strength+HIIT training can improve the overall sleep quality of sedentary adults, and there is no significant difference between the two (Jurado-Fasoli et al., 2020). However, other studies have shown that compared with strength training, aerobic training has a more significant effect on improving sleep quality in obese adults with chronic insomnia (Al-Jiffri and Abd El-Kader, 2021). The reason for the difference between this result and this study may be due to the different research objects. Considering that the subjects of the above studies are obese adults with insomnia, obesity will affect respiratory efficiency during sleep, and increase the risk of insomnia (Singareddy et al., 2012), which affects the overall sleep quality. Compared with strength training, aerobic training has greater energy consumption for obese people, a more obvious weight loss effect, and can indirectly improve sleep quality. Therefore, it is more effective in improving the sleep quality of obese chronic insomnia people. Therefore, whether exercise intervention can improve the overall sleep quality of different age and physical condition groups remains to be further studied.

### 4.2 Effect of different physical training programs on the improvement and regulation of PSQI components

Adolescents are in the process of growth and development, all sleep components are also changing (Vandendriessche et al., 2023). Severe sleep problems can be complicated by obesity, hypertension, diabetes, and other related diseases, which will affect academic performance (Kinoshita et al., 2021; Bugueño et al., 2017). Therefore, in the process of cultivating high school students, sleep health problems should be paid more attention to. In this study, after 12 weeks of training intervention, there was no significant change in the scores of other factors such as sleep quality, sleep latency, sleep duration, sleep disturbance, and daytime dysfunction of students who underwent traditional training, which was consistent with the trend of ordinary high school students. However, after training, the sleep latency and total sleep time components of the strength training group, the HIIT group, and the strength +HIIT group were significantly improved, and better than the traditional training group. This is consistent with the research of Kovacevic et al. (2018), Amara et al. (2020), and Jiménez-García et al. (2021). In addition, because the training volume and intensity of the strength+HIIT program are the sum of the alone strength training program and the HIIT program, the training intensity is relatively large, and the energy consumption is greater, so the group’s sleep disturbance score after training is significantly reduced, which is consistent with the study of Singh et al. (1997).

Although the strength group did not show an obvious effect after the intervention, it played a role in maintaining and regulating sleep, better than the traditional training group. It is concluded that the alone strength training program is more effective than the traditional training program in regulating sleep. While, in the regulation of sleep disorders, the strength+HIIT training program has a more positive impact. In terms of sleep latency and sleep duration, strength training program, HIIT program, and strength+HIIT program are more effective than traditional training programs.

In this study, the overall sleep level of the tested high school students is generally stable, but for the decline of the overall sleep quality of individual students, attention should be paid to and solved to prevent the emergence and development of sleep health problems and to avoid the impact of sleep health problems on academic performance, daily life, and even severe health problems.

### 4.3 Effect of different physical training programs on academic performance

In the Chinese education and talent evaluation system, academic performance, as an important measure of students’ learning ability, is one of the most fair and effective standards for their further education (Chen et al., 2021; Duan et al., 2018), especially for high school students. For a long time, academics have carried out a lot of discussions on the factors affecting students’ academic performance. Many studies have shown that academic performance is the result of the joint action of school, family, society, individual, and other factors (Gallardo et al., 2023; Rajendran et al., 2022; Affuso et al., 2023; Suleiman et al., 2024), among which individual is the most core factor. As an important means to promote the development of individual physical and mental health, physical exercise also has an important impact on students’ academic performance (Guimarães et al., 2023; Zurc and Planinšec, 2022; Zhang et al., 2023). According to domestic and foreign research, sports will not hinder the improvement of student’s academic performance but will play a positive role in promoting (Guimarães et al., 2023; Li et al., 2023). Relevant research in China has found that there is an intermediary mechanism between physical exercise and academic performance and encourages the improvement of academic performance through intermediary factors (Dong and Zhu, 2020; Park et al., 2023; Kayani et al., 2018; Muntaner-Mas et al., 2022; Fang, 2020).

In this study, after 12 weeks of physical training intervention, the academic performance of each group showed an increasing trend, especially in the strength+HIIT group, which had a positive effect on improving the academic performance of high school students. The results showed that there was no statistical significance between the growth changes of each group, Group IV (15.16) > Group III (11.58) > Group II (10.00) > Group I (5.30). However, except for the traditional group, the average growth values of the other three groups are all greater than 10. In the Chinese examination-oriented education evaluation system, especially for high school students, the increase of 10 points in academic performance will exceed thousands of people in the college entrance examination (Lifeng, 2010). Therefore, physical training may be a crucial method to improve academic performance. Studies have shown that both strength training and aerobic training can improve cognitive ability (Robinson et al., 2023; Tottori et al., 2019; Costigan et al., 2016), and both strength and aerobic fitness are positively correlated with cognitive ability and academic performance (Park et al., 2023; García-Hermoso et al., 2017; Peña et al., 2019). In addition, academic performance is an important manifestation of students’ cognitive ability. Therefore, the enhancement of cognitive ability can improve students’ learning ability and academic performance. However, the dose-effect relationship between physical activity and academic performance is related to many factors, such as training time, training frequency, training methods, etc. (Donnelly et al., 2016). In this study, the academic performance of the strength+HIIT group was significantly improved after training, probably because the training intensity, training time and training volume of this group were higher than those of other groups, which was consistent with the results of Bai et al. (2020).

Although most studies support the conclusion that moderate physical exercise can improve adolescents’ cognitive ability and academic performance (Wassenaar et al., 2020). However, the mechanism of physical exercise affecting adolescents’ academic performance needs further study (Wassenaar et al., 2020; Xu, 2015). Studies have shown that physical exercise can change the structure and function of the brain, increase the degree of brain activation and connectivity in the resting state, and then improve cognitive ability (such as memory, execution, etc.), thereby improving academic performance, but the research conclusions have not yet reached agreement (Zhou et al., 2023; Gunnell et al., 2019). Therefore, whether there is an intermediary variable between physical exercise and academic performance is discussed by scholars. Tomporowski et al. (2011) systematically summarized the mechanism of physical exercise affecting academic performance based on previous research, and used the model to express the mechanism. According to the model, the path of physical exercise affecting adolescents’ academic performance is more complex and has a direct impact. At the same time, it can also be affected by physical fitness, health, psychosocial and other intermediary factors. Among the health factors, sleep problems are included. Zhang et al. (2016) conducted a survey of 224 high school students and found that physical activity improves academic performance by improving sleep, and sleep plays an intermediary role between them. In this study, different training programs have different effects on the total quality of sleep, resulting in changes in academic performance. Among them, compared with other programs, strength+HIIT has a more significant positive effect on sleep and academic performance.

### 4.4 Limitations

The current study has some limitations that should be considered. First, our sample size was relatively small, which did not provide enough strength of value for detecting a difference among the groups. In addition, as the female sample was not included in the current study, so the results may not be generalized to this gender. Future studies with larger sample sizes and more female participants are warranted. Second, we only used subjective measures (PSQI) of sleep quality, duration, and daytime sleepiness. Future studies are encouraged to use objective techniques (i.e., polysomnography) to investigate the effects of different training programs on sleep architecture. Third, the study’s single-region design limits our ability to disentangle regional effects from intervention-specific outcomes. Replication in diverse geographical settings is critical to confirm the robustness of our conclusions. Future multi-regional RCTs are needed to evaluate whether our results hold across diverse socioeconomic, climatic, and cultural contexts. Finally, we did not consider the exercise volume of training programs. Further studies that assess physical activity using accelerometers and maximum oxygen consumption per unit of time are required to determine the dose–response relationship between physical activity amount and the magnitude of the effect on sleep and academic performance. This will contribute to uncovering what aspect of physical activity influences students’ sleep and academic achievement.

## 5 Conclusion

The overall sleep quality of senior high school students with traditional training is relatively stable, and its change trend is similar to that of ordinary senior high school students, that is, the traditional training program has no obvious regulation and improvement effect on the overall sleep quality of senior high school students. Compared with the traditional training program, strength training, HIIT and strength +HIIT training programs have more obvious effects on improving the overall sleep quality, mainly reflected in the two aspects of sleep latency and sleep duration. Secondly, the strength+HIIT program has a positive impact on improving sleep disturbance in high school students. In the use of sleep medication, traditional training not only failed to achieve a stable and improved effect but also showed a worsening trend, while strength training showed a positive improvement compared with traditional training. In addition, all four training programs can improve students’ academic performance, among which the strength+HIIT training program has a more noticeable effect.

It is suggested that high schools can consider the strength+HIIT concurrent training program when promoting and applying physical training, and pay attention to students’ sleep health problems to ensure that students have good sleep, which in turn ensures physical fitness and academic performance. Secondly, future research suggests that tracking and follow-up should be set up, and other indicators such as psychological situation and physical fitness should be combined to further optimize the physical training program for high school students.

## Data Availability

The original contributions presented in the study are included in the article/Supplementary material, further inquiries can be directed to the corresponding author.

## References

[ref1] Affuso G. Zannone A. Esposito C. Pannone M. Miranda M. C. De Angelis G.. (2023). The effects of teacher support, parental monitoring, motivation and self-efficacy on academic performance over time. Eur. J. Psychol. Educ. 38, 1–23. doi: 10.1007/s10212-021-00594-6

[ref2] Ait-Aoudia M. Levy P. P. Bui E. Insana S. De Fouchier C. Germain A.. (2013). Validation of the French version of the Pittsburgh sleep quality index addendum for posttraumatic stress disorder. Eur. J. Psychotraumatol. 4:19298. doi: 10.3402/ejpt.v4i0.19298, PMID: 24044071 PMC3773169

[ref3] Albrektsen M. Vestergaard T. H. Andreasen J. Nilsson K. K. (2021). A comparison of mental health and intelligence in young women and men eligible for military training. Personal. Individ. Differ. 170:110414. doi: 10.1016/j.paid.2020.110414

[ref4] Al-Jiffri O. H. Abd El-Kader S. M. (2021). Aerobic versus resistance exercises on systemic inflammation and sleep parameters in obese subjects with chronic insomnia syndrome. Afr. Health Sci. 21, 1214–1222. doi: 10.4314/ahs.v21i3.30, PMID: 35222584 PMC8843261

[ref5] Amara A. W. Wood K. H. Joop A. Memon R. A. Pilkington J. Tuggle S. C.. (2020). Randomized, controlled trial of exercise on objective and subjective sleep in Parkinson's disease. Mov. Disord. 35, 947–958. doi: 10.1002/mds.28009, PMID: 32092190 PMC8826749

[ref6] Amos L. Kuhn E. Kump T. Wade T. Grekowicz M. Bandla H.. (2019). The effect of consistent exercise on sleep in adolescents with sleep problems. Sleep 42:A303. doi: 10.1093/sleep/zsz067.752

[ref7] Astill R. G. Verhoeven D. Vijzelaar R. L. Van Someren E. J. (2013). Chronic stress undermines the compensatory sleep efficiency increase in response to sleep restriction in adolescents. J. Sleep Res. 22, 373–379. doi: 10.1111/jsr.12032, PMID: 23398048

[ref8] Bai S. Pan Z. Teng H. (2020). An empirical study of the impact of exercise on academic performance in middle school students. China Sport Sci. 40:9. doi: 10.16469/j.css.202011007

[ref9] Baranwal N. Phoebe K. Y. Siegel N. S. (2023). Sleep physiology, pathophysiology, and sleep hygiene. Prog. Cardiovasc. Dis. 77, 59–69. doi: 10.1016/j.pcad.2023.02.005, PMID: 36841492

[ref10] Bugueño M. Curihual C. Olivares P. Wallace J. López-Alegría F. Rivera-López G.. (2017). Quality of sleep and academic performance in high school students. Rev. Med. Chile 145, 1106–1114. doi: 10.4067/s0034-98872017000901106, PMID: 29424396

[ref11] Buysse D. J. Reynolds C. F. 3rd Monk T. H. Berman S. R. Kupfer D. J. (1989). The Pittsburgh sleep quality index: a new instrument for psychiatric practice and research. Psychiatry Res. 28, 193–213. doi: 10.1016/0165-1781(89)90047-4, PMID: 2748771

[ref12] Carek P. J. Laibstain S. E. Carek S. M. (2011). Exercise for the treatment of depression and anxiety. Int. J. Psychiatry Med. 41, 15–28. doi: 10.2190/Pm.41.1.c, PMID: 21495519

[ref13] Carpenter J. S. Andrykowski M. A. (1998). Psychometric evaluation of the Pittsburgh sleep quality index. J. Psychosom. Res. 45, 5–13. doi: 10.1016/s0022-3999(97)00298-5, PMID: 9720850

[ref14] Chen T. Y. Chou Y. C. Tzeng N. S. Chang H. A. Kuo S. C. Pan P. Y.. (2015). Effects of a selective educational system on fatigue, sleep problems, daytime sleepiness, and depression among senior high school adolescents in Taiwan. Neuropsychiatr. Dis. Treat. 11, 741–750. doi: 10.2147/ndt.S77179, PMID: 25834449 PMC4372029

[ref15] Chen Y. Huang R. Lu Y. Zhang K. (2021). Education fever in China: Children’s academic performance and parents’ life satisfaction. J. Happiness Stud. 22, 927–954. doi: 10.1007/s10902-020-00258-0

[ref16] Costigan S. A. Eather N. Plotnikoff R. C. Hillman C. H. Lubans D. R. (2016). High-intensity interval training for cognitive and mental health in adolescents. Med. Sci. Sports Exerc. 48, 1985–1993. doi: 10.1249/mss.0000000000000993, PMID: 27187097

[ref17] Courneya K. S. Segal R. J. Mackey J. R. Gelmon K. Friedenreich C. M. Yasui Y.. (2014). Effects of exercise dose and type on sleep quality in breast cancer patients receiving chemotherapy: a multicenter randomized trial. Breast Cancer Res. Treat. 144, 361–369. doi: 10.1007/s10549-014-2883-0, PMID: 24554388

[ref18] Dong Y. Zhu C. (2020). A study on the influence of extra-curricular sports on academic performance of teenagers: on the mediating effect of non-cognitive ability. J. Sports Res. 34:11. doi: 10.15877/j.cnki.nsic.20210108.005

[ref19] Donnelly J. E. Hillman C. H. Castelli D. Etnier J. L. Lee S. Tomporowski P.. (2016). Physical activity, fitness, cognitive function, and academic achievement in children: a systematic review. Med. Sci. Sports Exerc. 48, 1197–1222. doi: 10.1249/mss.0000000000000901, PMID: 27182986 PMC4874515

[ref20] Duan W. Guan Y. Bu H. (2018). The effect of parental involvement and socioeconomic status on junior school students’ academic achievement and school behavior in China. Front. Psychol. 9:952. doi: 10.3389/fpsyg.2018.00952, PMID: 29971025 PMC6018534

[ref21] Fang L. (2020). The effect of physical exercise on adolescents’ cognitive ability and academic achievements. China Sport Sci. 40:7. doi: 10.16469/j.css.202004004

[ref22] Faul F. Erdfelder E. Buchner A. Lang A. G. (2009). Statistical power analyses using G*power 3.1: tests for correlation and regression analyses. Behav. Res. Methods 41, 1149–1160. doi: 10.3758/brm.41.4.1149, PMID: 19897823

[ref23] Franklin B. A. Whaley M. H. Howley E. T. (2010). ACSM's guidelines for exercise testing and prescription. Medicine & Science in Sports and Exercise. 6th Edn. 4: 55. doi: 10.1046/j.1523-5408.2001.00105.x

[ref24] Gallardo L. O. Esteban-Torres D. Rodríguez-Muñoz S. Moreno-Doña A. Abarca-Sos A. (2023). Is there any relationship between physical activity levels and academic achievement? A cross-cultural study among Spanish and Chilean adolescents. Behav. Sci. 13:238. doi: 10.3390/bs13030238, PMID: 36975263 PMC10045662

[ref25] García-Hermoso A. Esteban-Cornejo I. Olloquequi J. Ramírez-Vélez R. (2017). Cardiorespiratory fitness and muscular strength as mediators of the influence of fatness on academic achievement. J. Pediatr. 187, 127–133.e3. doi: 10.1016/j.jpeds.2017.04.037, PMID: 28526219

[ref26] Gedda-Muñoz R. Fuentez Campos Á. Valenzuela Sakuda A. Retamal Torres I. Cruz Fuentes M. Badicu G.. (2023). Factors associated with anxiety, depression, and stress levels in high school students. Eur. J. Investig. Health Psychol. Educ. 13, 1776–1786. doi: 10.3390/ejihpe13090129, PMID: 37754468 PMC10529112

[ref27] Guimarães J. P. Fuentes-García J. P. González-Silva J. Martínez-Patiño M. J. (2023). Physical activity, body image, and its relationship with academic performance in adolescents. Healthcare 11:602. doi: 10.3390/healthcare11040602, PMID: 36833137 PMC9957426

[ref28] Gunnell K. E. Poitras V. J. Leblanc A. Schibli K. Barbeau K. Hedayati N.. (2019). Physical activity and brain structure, brain function, and cognition in children and youth: a systematic review of randomized controlled trials. Ment. Health Phys. Act. 16, 105–127. doi: 10.1016/j.mhpa.2018.11.002

[ref29] Guo R. Sun M. Zhang C. Fan Z. Liu Z. Tao H. (2021). The role of military training in improving psychological resilience and reducing depression among college freshmen. Front. Psych. 12:641396. doi: 10.3389/fpsyt.2021.641396, PMID: 34079481 PMC8166047

[ref30] Haltom R. W. Kraemer R. R. Sloan R. A. Hebert E. P. Frank K. Tryniecki J. L. (1999). Circuit weight training and its effects on excess postexercise oxygen consumption. Med. Sci. Sports Exerc. 31, 1613–1618. doi: 10.1097/00005768-199911000-00018 10589865

[ref31] Huang Y. S. Wang C. H. Guilleminault C. (2010). An epidemiologic study of sleep problems among adolescents in North Taiwan. Sleep Med. 11, 1035–1042. doi: 10.1016/j.sleep.2010.04.009, PMID: 20724214

[ref32] Hughes B. M. Steffen P. R. Thayer J. F. (2018). The psychophysiology of stress and adaptation: models, pathways, and implications. Int. J. Psychophysiol. 131, 1–3. doi: 10.1016/j.ijpsycho.2018.06.003, PMID: 29964069

[ref33] Hysing M. Harvey A. G. Linton S. J. Askeland K. G. Sivertsen B. (2016). Sleep and academic performance in later adolescence: results from a large population-based study. J. Sleep Res. 25, 318–324. doi: 10.1111/jsr.12373 26825591

[ref34] Jiménez-García J. D. Hita-Contreras F. De La Torre-Cruz M. J. Aibar-Almazán A. Achalandabaso-Ochoa A. Fábrega-Cuadros R.. (2021). Effects of HIIT and MIIT suspension training programs on sleep quality and fatigue in older adults: randomized controlled clinical trial. Int. J. Environ. Res. Public Health 18:1211. doi: 10.3390/ijerph18031211, PMID: 33572909 PMC7908512

[ref35] Jurado-Fasoli L. De-La O. A. Molina-Hidalgo C. Migueles J. H. Castillo M. J. Amaro-Gahete F. J. (2020). Exercise training improves sleep quality: a randomized controlled trial. Eur. J. Clin. Investig. 50:e13202. doi: 10.1111/eci.13202 31989592

[ref36] Kansagra S. (2020). Sleep disorders in adolescents. Pediatrics 145, S204–s209. doi: 10.1542/peds.2019-2056I, PMID: 32358212

[ref37] Kayani S. Kiyani T. Wang J. Zagalaz Sánchez M. L. Kayani S. Qurban H. (2018). Physical activity and academic performance: the mediating effect of self-esteem and depression. Sustain. For. 10:3633. doi: 10.3390/su10103633

[ref38] Kinoshita Y. Itani O. Otsuka Y. Matsumoto Y. Nakagome S. Osaki Y.. (2021). A nationwide cross-sectional study of difficulty waking up for school among adolescents. Sleep 44:zsab157. doi: 10.1093/sleep/zsab157, PMID: 34159386

[ref39] Klika B. Jordan C. (2013). High-intensity circuit training using body weight: maximum results with minimal investment. ACSMs Health Fit J 17, 8–13. doi: 10.1249/Fit.0b013e31828cb1e8

[ref40] Konjarski M. Murray G. Lee V. V. Jackson M. L. (2018). Reciprocal relationships between daily sleep and mood: a systematic review of naturalistic prospective studies. Sleep Med. Rev. 42, 47–58. doi: 10.1016/j.smrv.2018.05.005 30404728

[ref41] Kovacevic A. Mavros Y. Heisz J. J. Fiatarone Singh M. A. (2018). The effect of resistance exercise on sleep: a systematic review of randomized controlled trials. Sleep Med. Rev. 39, 52–68. doi: 10.1016/j.smrv.2017.07.002, PMID: 28919335

[ref42] Krishnan V. Collop N. A. (2006). Gender differences in sleep disorders. Curr. Opin. Pulm. Med. 12, 383–389. doi: 10.1097/01.mcp.0000245705.69440.6a, PMID: 17053485

[ref43] Landen S. Hiam D. Voisin S. Jacques M. Lamon S. Eynon N. (2023). Physiological and molecular sex differences in human skeletal muscle in response to exercise training. J. Physiol. 601, 419–434. doi: 10.1113/Jp279499, PMID: 34762308

[ref44] Li D. Wang D. Zou J. Li C. Qian H. Yan J.. (2023). Effect of physical activity interventions on children's academic performance: a systematic review and meta-analysis. Eur. J. Pediatr. 182, 3587–3601. doi: 10.1007/s00431-023-05009-w, PMID: 37227500

[ref45] Lifeng L. (2010). An analysis of the evolution and causes of the score line of college entrance examination——on the regional distribution of higher education entrance opportunities. Peking University Education Review 15, 56–70. doi: 10.3969/j.issn.1671-9468.2010.02.006

[ref46] Lim D. C. Najafi A. Afifi L. Bassetti C. L. Buysse D. J. Han F.. (2023). The need to promote sleep health in public health agendas across the globe. Lancet Public Health 8, e820–e826. doi: 10.1016/S2468-2667(23)00182-2, PMID: 37777291 PMC10664020

[ref47] Liu J. Ji X. Pitt S. Wang G. Rovit E. Lipman T.. (2024). Childhood sleep: physical, cognitive, and behavioral consequences and implications. World J. Pediatr. 20, 122–132. doi: 10.1007/s12519-022-00647-w, PMID: 36418660 PMC9685105

[ref48] Liu X. Tang M. (1996). Reliability and validity of the Pittsburgh sleep quality index. Chin. J. Psychiatr. 29:5.

[ref49] Lloyd R. S. Faigenbaum A. D. Stone M. H. Oliver J. L. Jeffreys I. Moody J. A.. (2014). Position statement on youth resistance training: the 2014 international consensus. Br. J. Sports Med. 48, 498–505. doi: 10.1136/bjsports-2013-092952, PMID: 24055781

[ref50] Longlalerng K. Nakeaw A. Charawae A. E. Reantong P. Prangyim U. Jeenduang N. (2021). Effects of six weeks high-intensity interval training and resistance training in adults with obesity and sleep related breathing disorders. Sleep Sci 14, 41–48. doi: 10.5935/1984-0063.20200076, PMID: 34917272 PMC8663736

[ref51] Mahfouz M. S. Ali S. A. Bahari A. Y. Ajeebi R. E. Sabei H. J. Somaily S. Y.. (2020). Association between sleep quality and physical activity in Saudi Arabian university students. Nat. Sci. Sleep 12, 775–782. doi: 10.2147/nss.S267996, PMID: 33117013 PMC7585794

[ref52] Mak K. K. Lee S. L. Ho S. Y. Lo W. S. Lam T. H. (2012). Sleep and academic performance in Hong Kong adolescents. J. Sch. Health 82, 522–527. doi: 10.1111/j.1746-1561.2012.00732.x, PMID: 23061556

[ref53] Manzar M. D. Moiz J. A. Zannat W. Spence D. W. Pandi-Perumal S. R. Hussain M. E. (2015). Validity of the Pittsburgh sleep quality index in Indian university students. Oman Med. J. 30, 193–202. doi: 10.5001/omj.2015.41, PMID: 26171126 PMC4459159

[ref54] Mendelson M. Borowik A. Michallet A. S. Perrin C. Monneret D. Faure P.. (2016). Sleep quality, sleep duration and physical activity in obese adolescents: effects of exercise training. Pediatr. Obes. 11, 26–32. doi: 10.1111/ijpo.12015, PMID: 25727885

[ref55] Miletínová E. Bušková J. (2021). Functions of sleep. Physiol. Res. 70, 177–182. doi: 10.33549/physiolres.934470, PMID: 33676389 PMC8820572

[ref56] Morris C. J. Aeschbach D. Scheer F. A. (2012). Circadian system, sleep and endocrinology. Mol. Cell. Endocrinol. 349, 91–104. doi: 10.1016/j.mce.2011.09.003, PMID: 21939733 PMC3242827

[ref57] Muntaner-Mas A. Mazzoli E. Abbott G. Mavilidi M. F. Galmes-Panades A. M. (2022). Do physical fitness and executive function mediate the relationship between physical activity and academic achievement? An examination using structural equation modelling. Children 9:823. doi: 10.3390/children9060823, PMID: 35740760 PMC9221993

[ref58] Nakajima S. Okajima I. Sasai T. Kobayashi M. Furudate N. Drake C. L.. (2014). Validation of the Japanese version of the ford insomnia response to stress test and the association of sleep reactivity with trait anxiety and insomnia. Sleep Med. 15, 196–202. doi: 10.1016/j.sleep.2013.09.022, PMID: 24380783

[ref59] Park S. Chun H. Etnier J. L. Yun D. (2023). Exploring the mediating role of executive function in the relationship between aerobic fitness and academic achievement in adolescents. Brain Sci. 13:614. doi: 10.3390/brainsci13040614, PMID: 37190579 PMC10137220

[ref60] Peña J. Yanez C. Gomez C. Martin W. Castillo C. Granados J.. (2019). The relationship between strength and academic performance: a new reason to promote physical activity. Eur. J. Pub. Health 29, 542–543. doi: 10.1093/eurpub/ckz186.429

[ref61] Philippot A. Dubois V. Lambrechts K. Grogna D. Robert A. Jonckheer U.. (2022). Impact of physical exercise on depression and anxiety in adolescent inpatients: a randomized controlled trial. J. Affect. Disord. 301, 145–153. doi: 10.1016/j.jad.2022.01.011, PMID: 35007642

[ref62] Qu G. Liu H. Han T. Zhang H. Ma S. Sun L.. (2024). Association between adverse childhood experiences and sleep quality, emotional and behavioral problems and academic achievement of children and adolescents. Eur. Child Adolesc. Psychiatry 33, 527–538. doi: 10.1007/s00787-023-02185-w, PMID: 36869931 PMC9985439

[ref63] Rajendran S. Chamundeswari S. Sinha A. A. (2022). Predicting the academic performance of middle- and high-school students using machine learning algorithms. Soc. Sci. Humanit. Open 6:100357. doi: 10.1016/j.ssaho.2022.100357

[ref64] Richards K. C. Lambert C. Beck C. K. Bliwise D. L. Evans W. J. Kalra G. K.. (2011). Strength training, walking, and social activity improve sleep in nursing home and assisted living residents: randomized controlled trial. J. Am. Geriatr. Soc. 59, 214–223. doi: 10.1111/j.1532-5415.2010.03246.x, PMID: 21314643 PMC3124380

[ref65] Richardson C. E. Gradisar M. Short M. A. Lang C. (2017). Can exercise regulate the circadian system of adolescents? Novel implications for the treatment of delayed sleep-wake phase disorder. Sleep Med. Rev. 34, 122–129. doi: 10.1016/j.smrv.2016.06.010, PMID: 27546185

[ref66] Robinson K. Riley N. Owen K. Drew R. Mavilidi M. F. Hillman C. H.. (2023). Effects of resistance training on academic outcomes in school-aged youth: a systematic review and meta-analysis. Sports Med. 53, 2095–2109. doi: 10.1007/s40279-023-01881-6, PMID: 37466900 PMC10587249

[ref67] Safarzade S. Tohidinik H. (2019). The sleep quality and prevalence of sleep disorders in adolescents. J. Res. Health 9, 471–479. doi: 10.32598/jrh.9.6.471, PMID: 40170207

[ref68] Shin Y. O. Lee J. B. Min Y. K. Yang H. M. (2013). Heat acclimation affects circulating levels of prostaglandin E2, Cox-2 and orexin in humans. Neurosci. Lett. 542, 17–20. doi: 10.1016/j.neulet.2013.03.017, PMID: 23523649

[ref69] Singareddy R. Vgontzas A. N. Fernandez-Mendoza J. Liao D. Calhoun S. Shaffer M. L.. (2012). Risk factors for incident chronic insomnia: a general population prospective study. Sleep Med. 13, 346–353. doi: 10.1016/j.sleep.2011.10.033, PMID: 22425576 PMC3319648

[ref70] Singh N. A. Clements K. M. Fiatarone M. A. (1997). A randomized controlled trial of progressive resistance training in depressed elders. J. Gerontol. A Biol. Sci. Med. Sci. 52, M27–M35. doi: 10.1093/gerona/52a.1.m27, PMID: 9008666

[ref71] Sletten T. L. Weaver M. D. Foster R. G. Gozal D. Klerman E. B. Rajaratnam S. M.. (2023). The importance of sleep regularity: a consensus statement of the National Sleep Foundation sleep timing and variability panel. Sleep Health 9, 801–820. doi: 10.1016/j.sleh.2023.07.016, PMID: 37684151

[ref72] Smith J. J. Beauchamp M. R. Faulkner G. Morgan P. J. Kennedy S. G. Lubans D. R. (2018). Intervention effects and mediators of well-being in a school-based physical activity program for adolescents: the 'Resistance training for Teens' cluster RCT. Ment. Health Phys. Act. 15, 88–94. doi: 10.1016/j.mhpa.2018.08.002, PMID: 40182660

[ref73] Spruit A. Assink M. Van Vugt E. Van Der Put C. Stams G. J. (2016). The effects of physical activity interventions on psychosocial outcomes in adolescents: a meta-analytic review. Clin. Psychol. Rev. 45, 56–71. doi: 10.1016/j.cpr.2016.03.006, PMID: 27064552

[ref74] Suleiman I. B. Okunade O. A. Dada E. G. Ezeanya U. C. (2024). Key factors influencing students’ academic performance. J. Electr. Syst. Inf. Technol. 11:41. doi: 10.1186/s43067-024-00166-w

[ref75] Tabata I. Nishimura K. Kouzaki M. Hirai Y. Ogita F. Miyachi M.. (1996). Effects of moderate-intensity endurance and high-intensity intermittent training on anaerobic capacity and Vo2max. Med. Sci. Sports Exerc. 28, 1327–1330. doi: 10.1097/00005768-199610000-00018, PMID: 8897392

[ref76] Taylor P. Heathcote A. Aidman E. (2021). Effects of multimodal physical and cognitive fitness training on subjective well-being, burnout and resilience in a military cohort. J. Sci. Med. Sport 24, S35–S36. doi: 10.1016/j.jsams.2021.09.092

[ref77] Tomporowski P. D. Lambourne K. Okumura M. S. (2011). Physical activity interventions and children's mental function: an introduction and overview. Prev. Med. 52 Suppl 1, S3–S9. doi: 10.1016/j.ypmed.2011.01.028, PMID: 21420981 PMC3160636

[ref78] Tottori N. Morita N. Ueta K. Fujita S. (2019). Effects of high intensity interval training on executive function in children aged 8-12 years. Int. J. Environ. Res. Public Health 16:4127. doi: 10.3390/ijerph16214127, PMID: 31717739 PMC6862681

[ref79] Uchida S. Shioda K. Morita Y. Kubota C. Ganeko M. Takeda N. (2012). Exercise effects on sleep physiology. Front. Neurol. 3:48. doi: 10.3389/fneur.2012.00048, PMID: 22485106 PMC3317043

[ref80] Vandendriessche A. Deforche B. Dhondt K. Altenburg T. M. Verloigne M. (2023). Combining participatory action research with intervention mapping to develop and plan the implementation and evaluation of a healthy sleep intervention for adolescents. Health Promot. Perspect. 13, 316–329. doi: 10.34172/hpp.2023.37, PMID: 38235009 PMC10790120

[ref81] Vartanian O. Fraser B. Saunders D. Suurd Ralph C. Lieberman H. R. Morgan C. A.. (2018). Changes in mood, fatigue, sleep, cognitive performance and stress hormones among instructors conducting stressful military captivity survival training. Physiol. Behav. 194, 137–143. doi: 10.1016/j.physbeh.2018.05.008, PMID: 29752975

[ref82] Visier-Alfonso M. E. Sánchez-López M. Álvarez-Bueno C. Ruiz-Hermosa A. Nieto-López M. Martínez-Vizcaíno V. (2022). Mediators between physical activity and academic achievement: a systematic review. Scand. J. Med. Sci. Sports 32, 452–464. doi: 10.1111/sms.14107, PMID: 34837413

[ref83] Vorster A. P. A. Van Someren E. J. W. Pack A. I. Huber R. Schmidt M. H. Bassetti C. L. A. (2024). Sleep health. Clinical and translational. Neuroscience 8:8. doi: 10.3390/ctn8010008, PMID: 40053772

[ref84] Wang G. Ren F. Liu Z. Xu G. Jiang F. Skora E.. (2016). Sleep patterns and academic performance during preparation for college entrance exam in Chinese adolescents. J. Sch. Health 86, 298–306. doi: 10.1111/josh.12379, PMID: 26930242

[ref85] Wassenaar T. M. Williamson W. Johansen-Berg H. Dawes H. Roberts N. Foster C.. (2020). A critical evaluation of systematic reviews assessing the effect of chronic physical activity on academic achievement, cognition and the brain in children and adolescents: a systematic review. Int. J. Behav. Nutr. Phys. Act. 17:79. doi: 10.1186/s12966-020-00959-y, PMID: 32571336 PMC7310146

[ref86] Xu W. (2015). Influence of physical exercise on cognitive function and academic performance in children: history, current status and future. China Sport Sci. 35:10. doi: 10.16469/j.css.2015.03.010

[ref87] Xu Z. Su H. Zou Y. Chen J. Wu J. Chang W. (2012). Sleep quality of Chinese adolescents: distribution and its associated factors. J. Paediatr. Child Health 48, 138–145. doi: 10.1111/j.1440-1754.2011.02065.x, PMID: 21470332

[ref88] Yeatts P. E. Martin S. B. Petrie T. A. (2017). Physical fitness as a moderator of neuroticism and depression in adolescent boys and girls. Personal. Individ. Differ. 114, 30–35. doi: 10.1016/j.paid.2017.03.040

[ref89] Yunus F. M. Ahmed M. S. Hossain M. B. Sarker K. K. Khan S. (2021). Gender variation of total sleep time and association with academic achievement among the school going adolescents: a cross-sectional study in rural Bangladesh. Sleep Epidemiol. 1:100001. doi: 10.1016/j.sleepe.2021.100001

[ref90] Zhang W. Jiao Y. Xu X. Yan J. (2016). Effects of high school students physical exercising on their academic achievements——mediating functions of sleep quality. J. Phys. Educ. 23, 135–140. doi: 10.16237/j.cnki.cn44-1404/g8.20161010.005

[ref91] Zhang Y. Yan J. Jin X. Yang H. Zhang Y. Ma H.. (2023). Sports participation and academic performance in primary school: a cross-sectional study in Chinese children. Int. J. Environ. Res. Public Health 20:3678. doi: 10.3390/ijerph20043678, PMID: 36834372 PMC9961712

[ref92] Zhou W. Saiz-González P. Rodriguez Aragon R. Adams K. Gao Z. (2023). Effect of physical activity interventions on brain structure and function changes in healthy children: a systematic review. Quest 76, 54–71. doi: 10.1080/00336297.2023.2217361

[ref93] Zhou H. Q. Shi W. B. Wang X. F. Yao M. Cheng G. Y. Chen P. Y.. (2012). An epidemiological study of sleep quality in adolescents in South China: a school-based study. Child Care Health Dev. 38, 581–587. doi: 10.1111/j.1365-2214.2011.01300.x, PMID: 21831260

[ref94] Zurc J. Planinšec J. (2022). Associations between physical activity and academic competence: a cross-sectional study among Slovenian primary school students. Int. J. Environ. Res. Public Health 19:623. doi: 10.3390/ijerph19020623, PMID: 35055444 PMC8775939

